# Selective attention on representations in working memory: cognitive and neural mechanisms

**DOI:** 10.7717/peerj.4585

**Published:** 2018-04-02

**Authors:** Yixuan Ku

**Affiliations:** Faculty of Education, East China Normal Unviersity, Shanghai, China; The Key Lab of Brain Functional Genomics, MOE & STCSM, Shanghai Changning-ECNU Mental Health Center, School of Psychology and Cognitive Science, East China Normal University, Shanghai, China; NYU-ECNU Institute of Brain and Cognitive Science, NYU Shanghai and Collaborative Innovation Center for Brain Science, Shanghai, China

**Keywords:** Attention orientation, Working memory, Object-based attention, Feature-based attention, Retrospective cue, Selective attention

## Abstract

Selective attention and working memory are inter-dependent core cognitive functions. It is critical to allocate attention on selected targets during the capacity-limited working memory processes to fulfill the goal-directed behavior. The trends of research on both topics are increasing exponentially in recent years, and it is considered that selective attention and working memory share similar underlying neural mechanisms. Different types of attention orientation in working memory are introduced by distinctive cues, and the means using retrospective cues are strengthened currently as it is manipulating the representation in memory, instead of the perceptual representation. The cognitive and neural mechanisms of the retro-cue effects are further reviewed, as well as the potential molecular mechanism. The frontal-parietal network that is involved in both attention and working memory is also the neural candidate for attention orientation during working memory. Neural oscillations in the gamma and alpha/beta oscillations may respectively be employed for the feedforward and feedback information transfer between the sensory cortices and the association cortices. Dopamine and serotonin systems might interact with each other subserving the communication between memory and attention. In conclusion, representations which attention shifts towards are strengthened, while representations which attention moves away from are degraded. Studies on attention orientation during working memory indicates the flexibility of the processes of working memory, and the beneficial way that overcome the limited capacity of working memory.

## Introduction

Working memory (WM) is a fundamental cognitive system that maintains and manipulates information from the outside world in a short period for goal-directed actions (Baddeley, 2012). WM is critical to support everyday behaviors including language comprehension, learning and reasoning (Baddeley, 2003). In spite of its core position in cognition, WM has severely limited capacity (Luck & Vogel, 2013). From the magic number seven (Miller, 1956) to the magic number four (Cowan, 2001), the limit in WM reflects the bottleneck of information processing in cognition. Researchers are fascinated about the mechanisms of WM capacity as the capacity is highly correlated with general intelligence (IQ) (Redick et al., 2011). Given the restricted resource of WM, it is essential to rely on selective attention, the goal-directed focus on certain aspects of the environment, while ignoring other irrelevant aspects. Empirical studies suggests that individual differences in WM capacity are correlated with the ability to control attention (Kane et al., 2001), and those who have lower WM capacity are not able to filter out distractors during WM maintenance (Vogel, McCollough & Machizawa, 2005). Therefore, effectively orienting attention during WM is important for goal-directed processes and behaviors.

## Survey Methodology

Since 1970s, research on WM and selective attention has increased exponentially (Baddeley, 2003; Baddeley, 2012; Carrasco, 2011). Searching through Web of Science (http://www.webofknowledge.com/) was used to identify the number of publications on both topics. As the two topics are both proper nouns, double quotation marks were used for precisely matched results. First, searching with topic of “working memory” from Web of Science between 1970 and 2016, there were 56,256 papers in total (See Data S1). Second, searching with topic of “selective attention” from Web of Science between 1970 and 2016, there were 14,214 papers in total (See  Data S1). Third, conjunction searching with topic of “working memory” AND topic of “selective attention” from Web of Science between 1970 and 2016, there were 2199 papers in total (See  Data S1). Searching in PubMed (http://www.ncbi.nlm.nih.gov) lead to similar results for the trends. First, searching with “working memory” in title or abstract (narrowed down from “topic”) from PubMed between 1970 and 2016, there were 22,930 papers in total. Second, searching with “selective attention” in title or abstract from PubMed between 1970 and 2016, there were 4,699 papers in total. Third, conjunction searching of the above two in title or abstract from PubMed between 1970 and 2016, there were 434 papers in total.

### Research on working memory and selective attention

**Figure 1:**
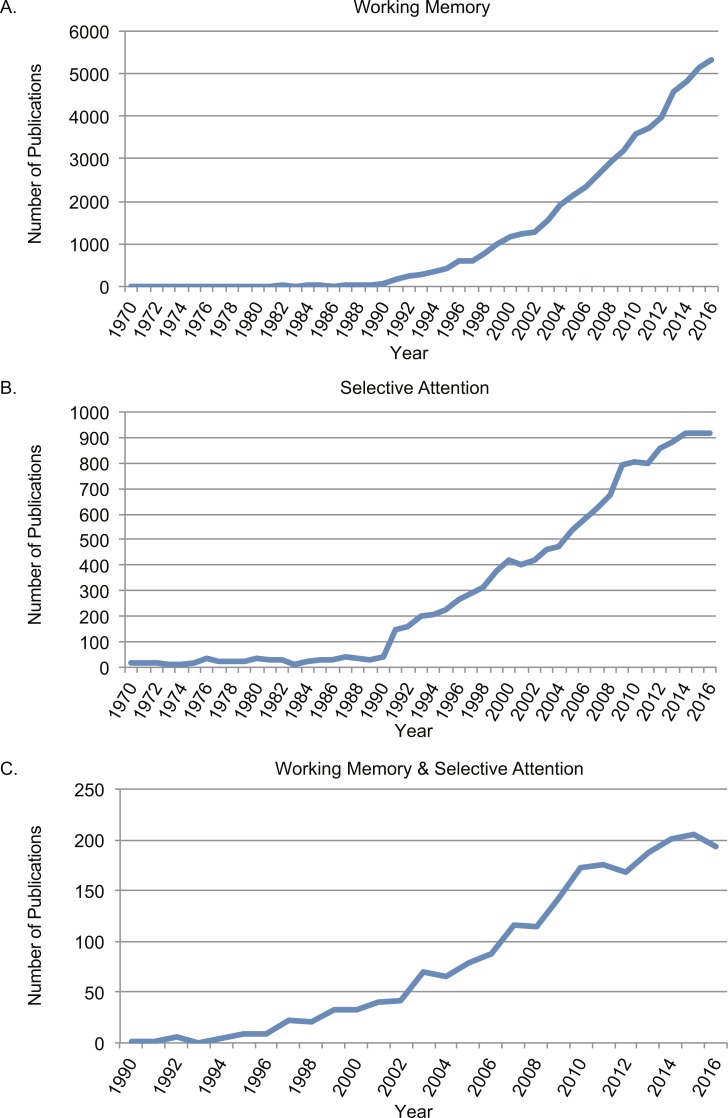
Number of publications searching from Web of Science between 1970 and 2016. (A) Searched results with topic in “working memory”; (B) searched results with topic in with topic in “selective attention”; (C) conjunction searched results with both “working memory” and “selective attention”.

The rapid increasing number of studies on WM and selective attention indicated that both of the concepts were within the focus of research interests on cognition. Figure 1 depicted searching results from Web of Science between 1970 and 2016 using topics of “working memory” (Fig. 1A), “selective attention” (Fig. 1B). The trend on both topics increased significantly after 1990s, when functional magnetic resonance imaging (fMRI) was developed and widely applied in research (Ogawa et al., 1990). The starting year in the conjunction search with both “working memory” and “selective attention” between 1970 and 2016 (Fig. 1C) was 1990 as well. It was increasingly acknowledged that selective attention and WM were inter-dependent cognitive functions (Awh, Vogel & Oh, 2006; Ku, 2015).

Based on the shared neural correlates between spatial WM and spatial selective attention (LaBar et al., 1999), Awh & Jonides (2001) proposed that the mechanisms of the two processes were overlapped. Some WM models even assumed that WM was merely representations from long-term memory that were under the focus of attention (Cowan, 1998). However, long-term memory plus attention model may not explain all WM processes as the representation in WM needs to integrates new incoming sensory information, and is much more flexible than that in long-term memory.

WM processes can influence selective attention, when high WM load introduces more distraction and less inhibition in selective attention (De Fockert et al., 2001). Meanwhile, attentional selection may also be guided by the template in WM (Downing, 2000; Soto et al., 2008). In contrary, selective attention can bias WM processing at multiple stages, from sensory encoding till memory retrieval, even during the stage prior to sensory stimuli, i.e., the expectation period (Gazzaley & Nobre, 2012). While traditionally the majority of studies focus on the influence of selective attention on perception (Carrasco, 2011), recent studies use retrospective cues to manipulate representations maintained in WM (Souza & Oberauer, 2016).

### Orienting attention during working memory maintenance by retrospective cues

Standard WM paradigm includes a stimulus (sample) that needs to remember, a short period (delay) when sample disappears and memorandums are maintained, the second stimulus (probe) for participants to judge whether it match the first one. Cues about the probed feature or spatial information could be presented at different stages: cues presented prior to the sample (sensory) stimuli are named *pre-cues*; cues presented when the sample stimuli are existing are named *sensory cues*; cues presented briefly after the sample stimuli disappeared are named *iconic cues*; cues presented after a couple of hundred milliseconds (usually 500 ms after the sample onset) are named *retro-cues*; cues presented after the probe stimuli are named *post-cues*. Different cues influence WM processes at distinctive stages as stated above.

Traditional work assumed that the performance of WM could only be affected during very short intervals after the offset of the sample stimuli, when the representations were thought as in an ‘iconic’ format, which had vast capacity (Phillips, 1974). After the representations were consolidated into WM, they became stable but then had limited capacity. Some theories further distinguished WM states as fragile vs. stable, based on the temporal progresses after sensory encoding (Sligte, Scholte & Lamme, 2008). The effects of iconic cues were very similar to those sensory cues during the sensory encoding period. Their effects were also similar to those pre-cues presented before the sample stimuli, which were originally introduced by Posner (1980). Seminar neuroimaging study by Kastner and colleagues in 1999 revealed that spatial attention induced by pre-cues changed the activity in human visual cortices during the expectation period (Kastner et al., 1999). The changed baseline neural activity would then biased the processing of incoming sensory information (Nobre & Van Ede, 2018). The pre-cues, sensory cues and iconic cues were thought to influence the perceptual representation, which might be different from the retro-cues that took impact on the representation in WM.

The retro-cue effects were first discovered independently by two groups (Landman, Spekreijse & Lamme, 2003; Griffin & Nobre, 2003), and they tended to be similar but a bit smaller than the pre-cue effects as the predictive cues before the sample stimuli influenced the perceptual processing that seemed to be more efficient than the memory processing (Griffin & Nobre, 2003). However, the processes on perception and memory representation were similar as reflected in ERP waveforms (Griffin & Nobre, 2003).

Retrospective cues could vary in different dimensions. First, there were spatial vs. feature/object cues (Li et al., 2015), which had similar effects. Second, there were also valid vs. invalid cues, when valid cues lead to better performance and invalid cues lead to worse performance (Gunseli et al., 2015). Third, the reliability of the cues could vary from 50% to 100%, while reliability increased the retro-cue effects (Shimi et al., 2013; Gunseli et al., 2015). Fourth, there were cues in different sensory domain, including visual (Landman, Spekreijse & Lamme, 2003; Griffin & Nobre, 2003), auditory (Backer & Alain, 2012) and tactile (Katus & Eimer, 2015), even crossmodal cues (Katus, Grubert & Eimer, 2016). Fifth, the retro-cue could be presented either centrally or peripherally, when the effects were comparable (Matsukura et al., 2014). Last, the time interval between the retro-cue and the probe could vary, and it was suggested that at least 300 ms were needed for the processes of retro-cue to take effects (Souza et al., 2014).

The distinctive effects induced by different retro-cues gave evidence for the flexibility of WM, indicating that more information could be extracted after the cue. Traditionally it was assumed that WM had fixed capacity limitations. It again suggested that long-term memory plus attention could not explain the processes of WM.

### Interference during working memory maintenance

Attention could be allocated towards the representations in WM and make additive enhancement to the task performance. On the other side, attention could also be directed away from WM representations by interference during the delay period, leading to worse task performance. There are two types of external interference, *distraction* (goal-irrelevant information that should be ignored) and *interruption* (information requiring attention as a secondary task). Both of them deteriorate WM performance, but to different extents and utilize distinct neural mechanisms (Clapp, Rubens & Gazzaley, 2010; Clapp et al., 2011; Clapp & Gazzaley, 2012). The filtering of distraction is thought to be dependent on top-down suppression signals from the prefrontal cortex (PFC) (Knight et al., 1999; Chadick, Zanto & Gazzaley, 2014), while an interruption requires a reallocation of cognitive resources, as well as processes involved in reactivating the disrupted representation afterwards, which is reliant on medial temporal lobe structures and the PFC (Sakai, Rowe & Passingham, 2002). Functional connectivity between stimulus-selective visual cortex and the prefrontal cortex, measured via functional magnetic resonance imaging (fMRI), has indicated that distraction does not change frontal-posterior functional connectivity during the delay, whereas interruptions result in a functional disconnection of the network that is reinstantiated after the interruption and prior to WM recall (Clapp, Rubens & Gazzaley, 2010).

Neural representation in the sensory cortices was thought to be fragile to interference and neural representation in the associative cortices (such as the prefrontal cortex, PFC and the posterior parietal cortex, PPC) was thought to be more stable (Bettencourt & Xu, 2015). The former and the latter were proposed to represent the quality and quantity of WM, respectively (Ku, Bodner & Zhou, 2015).

### Cognitive mechanism of attention orientation during working memory

Souza and Oberauer tested six hypotheses about the cognitive mechanisms underlying the retro-cue effect: (i) Protection from decay; (ii) Prioritizing for probe comparison; (iii) Enhancing the cued representations; (iv) Removing non-cued representations; (v) Affecting decision making processes; (vi) Protection from perceptual interference. The evidence discussed in their review provides support for the last four of these hypotheses (Souza & Oberauer, 2016).

It should be noted that there could be other cases of cognitive mechanisms underlying the retro-cue effect. Recently it was proposed that retro-cues first reoriented attention and then reconfigured the WM representation in the service of upcoming task demands (Myers, Stokes & Nobre, 2017). Besides, as attention was suggested to implement serially towards a spatial/temporal object in nature (Jia et al., 2017), the retro-cue might take effect through stabilizing the processes of attentional shift.

Since behavioral analysis could only show summarized experimental effects altogether, neural process along the entire temporal axis is critical to reveal the detailed dynamics of the mechanism. Future studies are still needed to make progresses on such neural mechanisms.

### Changing concepts of the neural mechanism underlying working memory

The concept of neural mechanism underlying WM has changed for a couple of times. Originally as the sustained delay activity in the prefrontal cortex was discovered to represent the mnemonic information (Fuster & Alexander, 1971), it was proposed that the prefrontal cortex was critical to maintain the representations of WM (Goldman-Rakic, 1995), the behavioral goals, as well as the means to achieve these goals (Miller & Cohen, 2001).

Afterwards, human neuroimaging studies further suggested the posterior parietal cortex as additional neural niche. Both fMRI and electroencephalography (EEG) revealed that the posterior neural activity changed with WM load and reached a plateau, which was similar to those behavioral findings (Todd & Marois, 2004; Vogel & Machizawa, 2004; Xu & Chun, 2006). However, both the behavioral and the neural plateau was recently challenged (Van den Berg & Ma, 2014; Bays, 2018).

The recent ten years witnessed the growing evidence that the posterior sensory regions were where the precise WM information is primarily stored (Ku, Bodner & Zhou, 2015; Christophel et al., 2017), and the role of the PFC was more emphasized in providing top-down control (D’Esposito et al., 1995; Smith & Jonides, 1999; Gazzaley & Nobre, 2012). Although traditional neuroimaging and neurophysiological studies indicated that the early sensory areas lacked of persistent delay activity (Bisley et al., 2004; Offen, Schluppeck & Heeger, 2009), the primary somatosensory cortex in rhesus monkey did show sustained and informative firing during the delay period of a tactile unimodal WM task (Zhou & Fuster, 1996).

Multivariate pattern analysis (MVPA) applied in neuroimaging data (Haynes & Rees, 2006; Davis & Poldrack, 2013) revealed that content-specific representations could be decoded during WM delay period from the primary visual areas (Serences et al., 2009; Harrison & Tong, 2009), as well as the primary auditory cortex (Kumar et al., 2016). Furthermore, visual motion patterns could not only be decoded from visual areas but also from the primary somatosensory cortex when the task is a pure visual WM task (Christophel & Haynes, 2014), suggesting the cross-modal modulation in the primary somatosensory cortex (Ku et al., 2007). The causal role of the primary sensory cortex in both unimodal and crossmodal WM were verified by recent transcranial magnetic stimulation (TMS) studies (Ku et al., 2015a; Ku et al., 2015b; Zhao et al., in press).

However, neurophysiological findings that the persistent modulation of activity in the primary visual cortex (Super, Spekreijse & Lamme, 2001) was argued to be feedback information from the associative cortices such as the PPC (Xu, 2017). The debating would be carried on unless there is solid evidence combing spatio-temporal neural recording and causal methods to manipulate the activity in the PFC or PPC, such as TMS or transcranial current stimulation (tCS).

### Neural mechanism of attention orientation during working memory

While filtering out distracting information is performed through directing attention away from distractors (Vogel, McCollough & Machizawa, 2005), attention orientation towards the target may implement similar mechanisms that the PFC controls accesses to WM (McNab & Klingberg, 2008; Reinhart et al., 2012). Different features of WM may involve distinctive frontal areas, since the frontal lobe could be divided into subdivisions based on the abstraction of processed goals (Badre, 2008).

**Figure 2:**
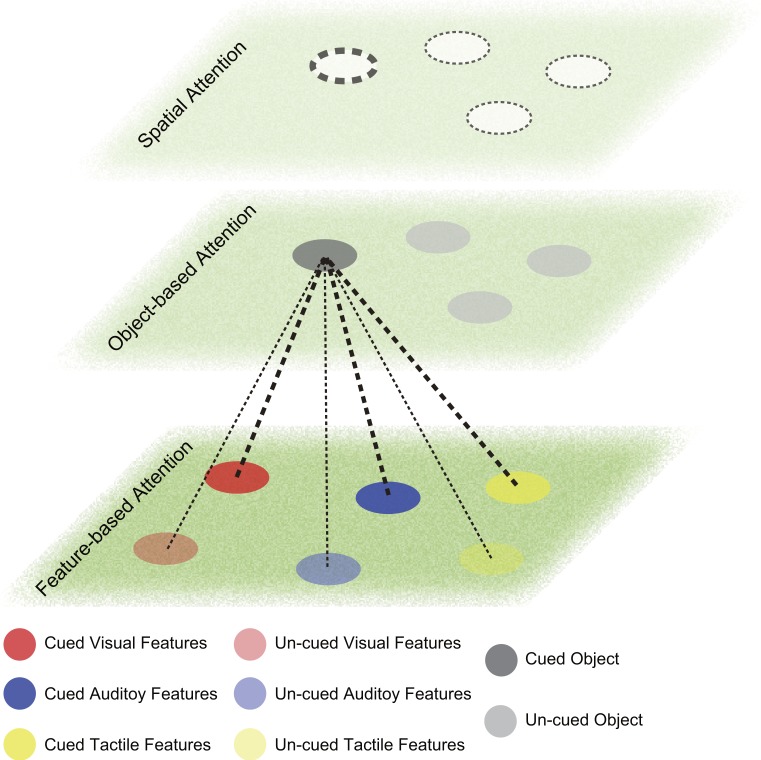
Attention orientation during working memory (WM). The lower plane is the feature-based attention space. Round patches with different colors indicate features represented in WM (red, visual; blue, auditory; yellow, tactile). The cued features are in dark color and the un-cued features are in light color. The middle plane is the object-based attention space. Grey round patches depict objects maintained in WM. Orienting attention to a cued object (darker grey) strengthen the representation of this object compared with other un-cued objects (lighter grey) in WM. It may strengthen some features connected to this object (thicker dashed lines), while other features remain (thinner dashed lines). The connection can be bi-directional, i.e. when attention is oriented to one feature; the object representation connected to this feature will be strengthened, but may not affect other feature representations from the same object. The upper plane is the spatial attention space. Dashed circles indicate attention allocation in the spatial map. The thicker circle indicates the prioritized focus of attention. The thinner circle indicates the divided focus of attention.

The neural dynamics of attention orientation during WM was revealed by EEG and Magnetoencephalography (MEG) studies. Most studies were within the visual domain and revealed both event related potentials (ERPs) and neural oscillations relating to the retro-cue effects (Kuo, Stokes & Nobre, 2012; Myers et al., 2015). MEG studies also confirmed the neural oscillations for spatial attention orientation during WM maintenance (Worden et al., 2000). Backer, Binns & Alain (2015) used an auditory delayed matching-to-sample task and a visual retro-cue directing attention to either the spatial information or the semantic category of the auditory target. Similar ERPs and neural oscillations of EEG compared with those in the visual domain were able to explain the behavioral benefits and dissociate the feature-specific vs. object-specific processes of attention orientation during WM. The attention orientation during tactile WM task also showed similar neural dynamics (Katus & Eimer, 2015). Such findings of retro-cue effects in visual, auditory and tactile domains support the theory of amodal attention orientation during WM (Shinn-Cunningham, 2008). The illustration of the process is depicted in Fig. 2. However, it is still unknown whether the object-based or feature-based attention orientation are the same in different sensory domains.

Moreover, neuroimaging studies in the visual domain suggested that the fronto-parietal network exerted top-down control (Corbetta & Shulman, 2002), which might also influenced attention orientation during WM as the retro-cues activated similar brain networks (Lepsien et al., 2005).

It should be noted that the fate of uncued item in WM was still debating. Some studies suggested that the uncued representations were removed out of the memory buffer or degraded at the cost of enhancing the cued item (Matsukura, Luck & Vecera, 2007; Kuo, Stokes & Nobre, 2012), while others indicate that they remained unaffected (Rerko & Oberauer, 2013). Future studies using MVPA with EEG/MEG may help resolve these arguments, by looking into the dynamic changes of the representation in WM.

Meanwhile, the development of the theory in attention orientation during WM will facilitate our understanding of the direction of information transfer between the sensory areas and the associative cortices during WM, which can be either feedforward or feedback (Corbetta & Shulman, 2002; Xu, 2017). Recent neurophysiological findings have revealed that the feedforward processing may rely on the gamma oscillation (40–90 Hz) and the feedback processing may react through alpha/beta (8–30 Hz) (Van Kerkoerle et al., 2014; Bastos et al., 2015; Van Kerkoerle, Self & Roelfsema, 2017). Combining neural modulation method (TMS or tCS) with neurophysiological recordings will help to validate these hypotheses.

Besides these neuronal mechanisms, rare molecular mechanisms were discovered for attention orientation during WM. Studies with animals have shown that dopamine D1 receptor in the prefrontal cortex is key in regulating WM, but not attention orientation in saccadic searching (Sawaguchi & Goldman-Rakic, 1991). Positron emission tomography (PET) study further reveal that within the prefrontal cortex dopamine D2 release is also more prominent in WM than sustained attention task (Aalto, 2005). On the other side, Psilocybin, a serotonin (5-HT) receptor agonist, affects attentional tracking task but not WM task (Carter et al., 2005). Therefore, dopamine may influence WM more than attention, and serotonin may take stronger effect on the other way. It would be interesting to see how these two neural transmitters interact with each other during attention orientation in WM, which needs elegant experimental design with animals.

## Conclusions

Selective attention and working memory interact with each other and share similar neural mechanisms. Using retrospective cues during WM is an efficient way to overcome the limited capacity of WM. The cued representations are strengthened, while the fate of the un-cued representations are still in debate, either degraded or unchanged. Future studies implementing both neural modulation methods (TMS and tCS) and neurophysiological recording (EEG/MEG) are critical to consolidate the existing hypotheses and to help resolve the controversial theories in this expanding field.

##  Supplemental Information

10.7717/peerj.4585/supp-1 Data S1 The raw data to generate Figure 1 Numbers of publications searching from the Web of Science between 1970 and 2016 using topic of “working memory”, or “selective attention”, or conjunction search of the two.

## Additional Information and Declarations

### Data Availability

The following information was supplied regarding data availability: The raw data are provided in a Data S1.
